# Breeding for the absence of proanthocyanidins in grain of barley (Hordeum vulgare L.): molecular genetic and technological aspects

**DOI:** 10.18699/vjgb-25-142

**Published:** 2025-12

**Authors:** C.A. Molobekova, I.V. Totsky, N.V. Trubacheeva, O.Yu. Shoeva

**Affiliations:** Institute of Cytology and Genetics of the Siberian Branch of the Russian Academy of Sciences, Novosibirsk, Russia; Institute of Cytology and Genetics of the Siberian Branch of the Russian Academy of Sciences, Novosibirsk, Russia; Institute of Cytology and Genetics of the Siberian Branch of the Russian Academy of Sciences, Novosibirsk, Russia; Institute of Cytology and Genetics of the Siberian Branch of the Russian Academy of Sciences, Novosibirsk, Russia

**Keywords:** barley, grain, proanthocyanidins, beer haze, proanthocyanidin-free, brewing, ячмень, зерно, проантоцианидины, коллоидное помутнение, беспроантоцианидиновый, пивоварение

## Abstract

Phenolic compounds constitute a significant group of secondary metabolites in barley grain and influence its technological qualities when used in brewing, feed production, and food manufacturing. Proanthocyanidins – polymeric flavonoids localized in the seed coat – play a particularly important role among them. These compounds are responsible for several production issues, such as colloidal haze in beer and browning of groats after heat treatment. Although proanthocyanidins possess health-beneficial properties based on their antioxidant activity, they can act as antinutritional factors due to their ability to bind proteins. In this regard, the breeding of barley varieties completely lacking proanthocyanidins in the grain was initiated, primarily for use in the brewing industry. Initially, it was assumed that their absence would not be critical for the plant, since wheat, corn, and rice varieties lacking proanthocyanidins in the grain had been identified. However, accumulated evidence indicates that proanthocyanidins perform important physiological functions: they contribute to the maintenance of seed dormancy, provide protection against fungal and bacterial pathogens and pests, and their absence negatively affects agronomic traits. For instance, proanthocyanidin-free barley mutants obtained through induced mutagenesis exhibit reduced productivity and pathogen resistance, an increased risk of pre-harvest sprouting, and deterioration of several technologically important properties. Nevertheless, these mutant lines are actively used in breeding programs to develop varieties for various purposes. This review aims to systematize and analyze global experience in breeding proanthocyanidin-free barley varieties, describing achieved results to identify the most successful approaches and define future research directions. The work examines challenges faced by breeders when using mutant lines, as well as strategies that have helped minimize negative side effects. It is demonstrated that through targeted crossing and optimal selection of mutant alleles, competitive varieties have been developed that combine the required technological qualities with satisfactory agronomic performance, meeting the demands of both the brewing and food industries.

## Abstract (ru)

Фенольные соединения составляют значимую группу вторичных метаболитов зерна ячменя и оказывают влияние на его технологические качества при использовании в пивоварении, производстве кормов и пищевых продуктов. Особую роль среди них играют проантоцианидины – полимерные флавоноиды, локализованные в семенной оболочке. Эти соединения обусловливают ряд производственных проблем, таких как коллоидное помутнение пива, а также потемнение крупы после термообработки. Хотя проантоцианидины обладают полезными для здоровья человека свойствами, основанными на их антиоксидантной активности, они могут выступать как антипитательные факторы из-за своей способности связывать белки. В связи с этим была инициирована селекция сортов ячменя, полностью лишенных проантоцианидинов в зерне, в первую очередь для использования в пивоваренной промышленности. Первоначально предполагалось, что их отсутствие не критично для растения, поскольку у пшеницы, кукурузы и риса были выявлены образцы, не содержащие проантоцианидинов в зерне. Однако накопленные данные свидетельствуют, что проантоцианидины выполняют важные физиологические функции: участвуют в поддержании покоя семян, обеспечивают защиту от грибных и бактериальных патогенов и вредителей, и их отсутствие негативно сказывается на агрономических характеристиках. Так, у беспроантоцианидиновых мутантов ячменя, полученных методами индуцированного мутагенеза, отмечено снижение продуктивности и устойчивости к патогенам, повышение риска прорастания зерна на корню и ухудшение ряда технологически важных свойств. Тем не менее мутантные линии активно используются в селекционных программах для создания сортов различного назначения. Цель данного обзора – систематизировать и проанализировать мировой опыт селекции беспроантоцианидиновых сортов ячменя, описать достигнутые результаты для выявления наиболее успешных подходов и определения направлений дальнейших исследований. В работе рассматриваются проблемы, с которыми столкнулись селекционеры при использовании мутантных линий, а также стратегии, позволившие минимизировать негативные побочные эффекты. Показано, что за счет целенаправленного скрещивания и оптимального подбора мутантных аллелей удалось создать конкурентоспособные сорта, сочетающие требуемые технологические качества с удовлетворительными агрономическими характеристиками и отвечающие запросам как пивоваренной, так и пищевой промышленности.

## Introduction

Barley (Hordeum vulgare L.) is an important agricultural crop widely used in brewing, forage production, and the food industry. Its grain quality is determined not only by protein and starch content but also by secondary metabolites, particularly phenolic compounds, which accumulate in the grain husk (Van Hung, 2016). Among them, proanthocyanidins (PAs) are of particular interest due to their significant role in plant physiology. The deposition of PAs in the seed coat is associated with their role in maintaining seed dormancy (Debeaujon et al., 2000) and protecting the developing seed from various factors, including fungal and bacterial pathogens, insect pests, and heavy metal exposure (Dixon et al., 2005).

The nutritional and feed value of PAs is controversial. On the one hand, numerous studies describe their potentially beneficial properties for human health (Santos-Buelga, Scalbert, 2000). In animal husbandry, these compounds are being studied as a feed additive alternative to antibiotics (Kumar K. et al., 2022). In ruminants, a moderate amount of PAs in feed (2–4 %) can have a beneficial effect by reducing inefficient protein degradation in the rumen. On the other hand, PAs act as antinutritional factors when feeding monogastric animals, since their digestive system is unable to effectively digest PA complexes with proteins (Dixon et al., 2005). PA causes undesirable darkening when barley porridge is prepared, thereby negatively affecting the product’s consumer quality (Kohyama et al., 2009).

In the brewing industry, PAs are the main cause of colloidal haze, which occurs during cooling of beer and impairs its stability (Delcour et al., 1984). Proteases and selective absorbents are used to solve this problem; however, these methods are not specific enough and can affect the taste and quality of beer (Wang, Ye, 2021). A more effective approach is to develop barley cultivars with reduced levels of protein or PAs in the grain, which helps prevent haze without the use of stabilizers. Since reducing the protein fraction of grain can have a more harmful effect on the plant, breeding of proanthocyanidin-free (PA-free) cultivars is considered a priority (Erdal, 1986; von Wettstein, 2007). This became possible due to the creation of induced barley mutants with impaired flavonoid synthesis, which have served as donors of the target trait in breeding programs (Jende-Strid, 1993).

This review discusses the challenges and prospects associated with breeding PA-free barley cultivars. Since PAs are involved in the regulation of seed dormancy and stress protection, their absence may be accompanied by changes in grain germination rate, plant resistance to pathogens, malt modification, and other important traits. Understanding these relationships is essential for developing commercial barley cultivars that combine the absence of PAs with high agronomic and technical performance.

## Molecular and genetic basis of proanthocyanidin synthesis in barley

Flavonoid synthesis begins with the formation of chalcone by the condensation of three malonyl-CoA molecules with one 4-coumaroyl-CoA molecule. 4-Coumaroyl-CoA serves as a precursor not only for flavonoids but also for lignans, allomelanins, and lignin, which are also found in barley grain (Bartłomiej et al., 2012; Shoeva et al., 2020; Yu et al., 2023). The flavonoid synthetic pathway leads to the formation of leucoanthocyanidins, which are common precursors of PAs and anthocyanins (see the Figure). At this stage, the pathway branches: leucoanthocyanidin reductase (LAR) catalyzes the synthesis of flavan-3-ols, the monomers of PA, while the competitive enzyme anthocyanidin synthase (ANS) oxidizes leucoanthocyanidins to anthocyanidins (Saito et al., 1999; Tanner et al., 2003). Subsequent glycosylation of anthocyanidins leads to the formation of anthocyanins. Anthocyanidins can also be reduced to flavan-3-ols by anthocyanidin reductase (ANR), thus forming an alternative pathway for PA synthesis (Xie et al., 2003).

The polymerization of flavan-3-ols to form PAs probably occurs non-enzymatically, although the involvement of an unknown flavanol-condensing enzyme has been previously suggested (Jende-Strid, 1993; He et al., 2008; Yu et al., 2023). Flavan-3-ols are proposed as the starting units of polymerization, while leucoanthocyanidins and their derivatives are considered to be the extention units. Another important aspect of PA biosynthesis is their intracellular transport (see the Figure). Flavan-3-ols are synthesized in the cytosol, while the final site of PA accumulation is the vacuole (Winkel, 2004). The transport of flavan-3-ols into the vacuole is mediated by MATE family proteins, the functioning of which depends on the proton gradient on the vacuolar membrane generated by H+-ATPases (Yu et al., 2023). In addition, proteins of the glutathione-S-transferase (GST) family are involved in the transport of flavonoids, including PA, which presumably perform the function of binding and delivering flavonoids to vacuolar transporters (Pérez-Díaz et al., 2016). A tannosome model of PA synthesis was also proposed according to which PA polymerization occurs in specialized organelles – tannosomes, formed from chloroplast thylakoids (Brillouet et al., 2013).

PA biosynthesis is under complex transcriptional control. The key regulatory module is the MBW complex, consisting of transcription factors of the MYB, bHLH, and WD40 families (Bulanov et al., 2025). The complex activates the expression of structural genes of the flavonoid biosynthesis pathway, providing their spatiotemporal regulation. In barley, a MYB factor encoded by the HvMyb10 (or Ant28) gene has been characterized; it specifically controls PA synthesis by regulating the expression of Dfr and Lar genes (Jende- Strid, 1993; Himi et al., 2011). In addition to the MBW complex, transcription factors of the WRKY, MADS, and WIP families are involved in the regulation of PA synthesis (He et al., 2008).

Many genes involved in barley flavonoid synthesis were identified through analysis of the Anthocyanin-less (Ant) mutant collection. This collection was created in the 1970s through induced mutagenesis of various cultivars and lines from Europe, USA, and Japan and comprises about 900 mutants with impaired flavonoid synthesis. Based on allelism testing of 566 mutants, 30 Ant loci were described (Jende- Strid, 1993). To date, the molecular functions of only seven of them have been established (see the Figure). Of particular interest are the Ant19 and Ant25–Ant29 loci, mutations in which specifically suppress PA synthesis. Although the molecular functions for many loci remain unknown, a potential function has been proposed for some of them based on biochemical analysis of mutants. For example, the Ant25, Ant27, and Ant29 loci presumably encode regulatory factors, since the activity of several enzymes of the biosynthetic pathway is impaired in the corresponding mutants. In contrast, Ant26 is likely a structural gene controlling the final stages of synthesis, since ant26 mutant grains accumulate monomeric flavan-3-ols in the absence of PA (Jende-Strid, 1993).

Mutant lines not only became an important tool for studying PA synthesis genes but also formed the basis for the development of PA-free brewing cultivars. The first such line was ant13.13, obtained from the Foma cultivar. Despite the reduced yield of ant13.13 compared to the original cultivar, beer produced from the grain of this line demonstrated excellent colloidal stability (von Wettstein et al., 1977). Furthermore, using PA-free mutants, it was shown that the absence of PA in beer does not affect its organoleptic characteristics (von Wettstein et al., 1977; Delcour et al., 1984). These results confirmed the potential of this approach and stimulated further breeding work to develop PA-free barley cultivars, which, in addition to PA content, must meet the general requirements for malting.

## Quality parameters of brewing barley cultivars

Malting barley is subject to stringent requirements (GOST 5060–2021) that must be taken into account during selection. A key quality criterion is protein content, which should not exceed 11.5–12.0 % of dry weigh (DW). Excess protein inhibits starch degradation and reduces extractability, while a protein deficiency limits yeast nutrition. Suffitient starch content, which is converted into fermentable sugars, is equally important. For malting barley, starch content should be at least 60–65 % of DW (Golovin et al., 2008).

The grain nature (grain weight per liter) of malting barley, which characterizes its plumpness, must be no less than 660 g/L. The 1,000-kernel weight depends on the grain size and is optimal within the range of 40–50 g, as grains that are too large do not malt quickly enough. The grain size, determined by the proportion of grains passing through a 2.5×20 mm sieve, should be no less than 85 % for first-class malting barley, as larger grains contain more starch and have higher extractability. Husk content, or the percentage of husks in the total grain weight, should not exceed 9 %, as an excess husk reduces the starch content and extractability and impairs beer taste, although a moderate amount of husk is necessary for the formation of a filter layer (Khokonova, 2015). The moisture content of raw grain should not exceed 12 % to prevent mold growth and mycotoxin accumulation during storage (Chi et al., 2003). Germination energy and germination capacity are critical for uniform malting, as starch in ungerminated grains is not fully fermented, which reduces extract and beer yield. Good-quality grain should have a germination capacity of over 95 %.

A crucial stage in beer production is malting, during which a complex of hydrolytic enzymes (proteases and amylases) is formed. The resulting malt serves not only as a substrate for fermentation but also as a source of coloring and aromatic components for beer (Bamforth, 2009). The main quality indicator of malt is malt extract – the proportion of dry matter transferred to the wort – which is at least 79 % for high-quality grain. Diastatic power, measured in Windesch–Kolbach units (WK) and characterizing the activity of amylolytic enzymes, should exceed 220 WK units for spring barley and 350 WK units for winter barley. The Kolbach index is an indicator of the degree of protein degradation and is expressed as the ratio of soluble nitrogen to the total nitrogen content in the malt. Deviation from the optimal values of 35–49 % lead to filtration problems or taste deterioration (Kumar V. et al., 2023). Free amino nitrogen content is essential for normal yeast metabolism during fermentation. The optimal concentration is 140–180 mg/L. The β-glucan content in malt should not exceed 200 mg/100 g, as its excess increases wort viscosity, which impairs filtration and reduces beer quality. These indicators are quantitative traits and are polygenically controlled (Trubacheeva, Pershina, 2021), which significantly complicates barley breeding for brewing purposes.

## Advances in breeding proanthocyanidin-free barley cultivars

Developing PA-free barley cultivars that meet stringent brewing standards has been challenging because the original PA-free mutants exhibited a number of disadvantages, including low grain plumpness, reduced yield and 1,000-kernel weight (Figueroa et al., 1989; Bregitzer et al., 1995; Wu, 1995). Since PAs are involved in maintaining seed dormancy, reduced grain dormancy was observed in the mutants (Himi et al., 2011). Although accelerated and uniform germination may be an advantage for malting, reduced dormancy increases the risk of pre-harvest sprouting, especially under high humidity conditions.

Technological limitations were also of particular importance. Low protein content is desirable for brewing cultivars, but PA-free mutants tended to have higher protein content than the original cultivars (von Wettstein et al., 1977; Øverland et al., 1994; Wu, 1995). Malt produced from the mutant grain exhibited reduced malt extract, diastatic power, and degree of attenuation (Bregitzer et al., 1995). Despite these limitations, mutant lines were actively used in breeding of PA-free cultivars, which were registered and cultivated in Europe, USA, Japan, and the Republic of Korea (see the Table).

**Table 1.**
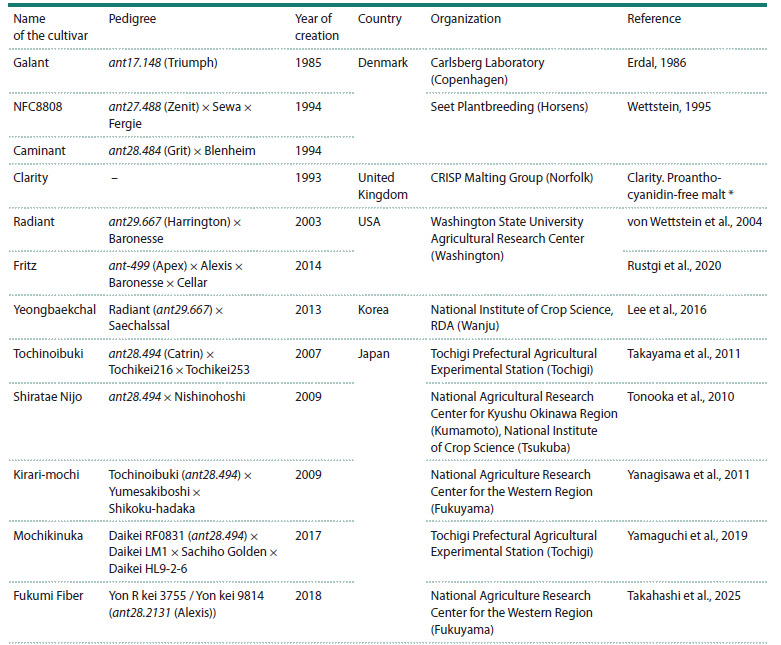
Registered proanthocyanidin-free barley cultivars Clarity. Proanthocyanidin-free malt. 1999. CRISP Malting Group. Available at: https://www.yumpu.com/en/document/read/4110511/

In Denmark, the Galant cultivar was bred from the ant17.148 mutant. In trials conducted in 1982–1983 in 13 European regions, Galant showed yield only 1–7 % lower than the standard (Erdal, 1986). However, Galant had reduced synthesis of enzymes that degrade cell wall polysaccharides and starch compared to the original cultivar, which led to a decrease in the proportion of fermentable sugars and an increase in wort viscosity (Palmer, 1988). The Japanese line Mokkei 92-130, derived from the ant13.347 mutant, was relatively successful, demonstrating high malt extract and diastatic power. However, the beer prepared from it was characterized by an accelerated deterioration of organoleptic properties (Fukuda et al., 1999), which was probably due to the absence of phenolic compounds that usually ensure oxidative stability of beer. In the USA, more than 40 promising PA-free lines were created based on the ant-517 mutant (Wesenberg et al., 1989). Many of these lines were equal to or superior to the standard Klages cultivar in most malting quality indicators, but were inferior in yield, percentage of malt extract, and also had an excessive amount of protein (Wesenberg et al., 1989). Also in the USA, PA-free lines with improved traits were created based on the ant13.582 line and the Azure, Glenn, and Hazen cultivars: earlier heading, increased roundness of grains and higher soluble protein content. However, due to the negative impact of the mutant ant13 allele on malt quality and other traits, the authors considered the ant13.582 line unsuitable for further breeding (Horsley et al., 1991).

Thus, practice has shown that mutations blocking the initial stages of PA biosynthesis are generally unsuitable for cultivar development due to their pleiotropic effects on yield or brewing quality. The use of mutants with impaired PA synthesis at the final stages is more promising, as it minimizes the side effects of mutations. For example, mutants at the Ant26 locus, which have impaired PA synthesis at the final stages, demonstrate productivity at the level of the original cultivar Grit (Totsky et al., 2024). In Denmark, the NFC8808 and Caminant cultivars were developed based on the ant27.488 and ant28.484 mutants, respectively. The Caminant cultivar exceeded the standard yield by 4 %, including in conditions where there was no fungicidal protection (Wettstein, 1995). It met the European Alexis standard in key parameters such as germination index, malt extract, diastatic power, nitrogen content, and β-glucan content. In 1999, the Clarity cultivar, characterized by high colloidal stability and yield, was registered in the United Kingdom (see the Table). The authors did not report the cultivar’s pedigree, but the presence of anthocyanin pigmentation in the vegetative organs of Clarity indicates that the synthesis was disrupted at the final steps.

It is worth noting that the breeding of feed and food PA-free cultivars is actively developing in addition to malting cultivars. In 2003, the Radiant cultivar, based on the ant29.667 mutant, was officially approved in the USA. Due to its heat-stable β-amylase, Radiant possessed high diastatic power (von Wettstein et al., 2004). The cultivar also demonstrated high yield, resistance to several pathogens, and improved nutritional characteristics, making it suitable for use not only in the brewing but also in the food industry. Korean breeders, based on Radiant, developed the food cultivar Yeongbaekchal (Lee et al., 2016). Porridge made from Yeongbaekchal grain did not darken after heat treatment, making the product more attractive to consumers. The cultivar was resistant to barley yellow mosaic virus (BaYMV), and its yield was only 5 % lower than the control.

In the USA, the Fritz cultivar, which exhibits excellent germination and high resistance to yellow and leaf rust and powdery mildew, was developed from the ant-499 mutant (Rustgi et al., 2020). The locus carrying the mutation in the original mutant ant-499 has not been determined. However, the presence of anthocyanins in the stem of Fritz indicates a defect in PA biosynthesis specifically at late stages. Although Fritz was registered as a forage cultivar due to its high protein and β-glucan content, it also demonstrated good malting qualities, making it a potential dual-purpose cultivar.

Japanese researchers developed the two-row Shiratae Nijo cultivar using the ant28.494 mutant (Tonooka et al., 2010). Despite the initial late maturity anf susceptibility of the ant28.494 mutant to BaYMV, they obtained a cultivar not inferior to the original Nishinohoshi in yield and resistance to BaYMV and powdery mildew. Notably, despite reduced levels of flavan-3-ols and PAs, compounds with antifungal activity, Shiratae Nijo’s susceptibility to fusarium head blight did not exceed that of the original cultivar. The same ant28.494 mutant was used to create the Tochinoibuki cultivar (Takayama et al., 2011), which is similar to the standard Sukai Golden in heading and ripening dates and 1,000-kernel weight. Subsequently, the Tochinoibuki cultivar was used to develop the naked, waxy, PA- and amylose-free Kirarimochi cultivar (Yanagisawa et al., 2011). Pearled barley from Kirari-mochi grain contained 1.5 times more β-glucans than the standard Ichibanboshi cultivar, making it particularly valuable for the production of functional food products. A waxy, PA-free Mochikinuka cultivar was also developed from Tochinoibuki. The absence of the enzyme lipoxygenase- 1, which catalyzes lipid oxidation, in this cultivar led to a more pleasant taste of the groats (Yamaguchi et al., 2019). A recent achievement of Japanese breeding was the six-row naked cultivar Fukumi Fiber, which combines the mutant alleles wax and amo1, which control the content of β-glucans, and ant28 (Takahashi et al., 2025). Due to this, the β-glucan content in Fukumi Fiber grain reached 13.2 % – three times higher than the standard Ichibanboshi and twice as high as the waxy Kirari-mochi.

Thus, despite the significant shortcomings inherent in PA- free mutants, targeted breeding work has proved the possibility of creating competitive barley cultivars based on them.

## Conclusion

Breeding malting barley cultivars that do not accumulate PA in grain has been associated with a number of challenges related to reduced yield and grain quality. However, based on world experience in developing PA-free cultivars, it can be concluded that the choice of a mutant line as a trait donor largely determines the success of breeding. It has been shown that mutations in genes specifically controlling the PA synthesis branch have the least negative impact on agronomic traits, as the synthesis of other physiologically important flavonoids is preserved. Based on breeding experience and comparative morphological studies, mutants for the Ant26, Ant28, and Ant29 genes currently represent the most promising donors for breeding PA-free cultivars. Despite the high breeding potential of mutants in these genes, the molecular functions of only one of them, Ant28, are known. The lack of data on the functions of the remaining genes makes it impossible to develop DNA markers for selection, which are effectively used in breeding for the Ant28 gene. This highlights the need for fundamental research into the molecular genetic mechanisms of PA biosynthesis, since a deep understanding of these fundamentals is the key to the creation of competitive commercial cultivars.

## Conflict of interest

The authors declare no conflict of interest.
